# Supplementary material for: CHEK2 Pathogenic Variants in Greek Breast Cancer Patients: Evidence for Strong Associations with Estrogen Receptor Positivity, Overuse of Risk-Reducing Procedures and Population Founder Effects
Source: Cancers (Basel). 2021 Apr 27;13(9):2106. doi: 10.3390/cancers13092106 (PMC8123864; doi:10.3390/cancers13092106)
Supplement: Supplementary file 1 [file cancers-13-02106-s001.zip › cancers-1186018-supplementary.pdf]

Table S1. Estimated allele frequencies of the microsatellite markers D22S1163, D22S689, D22S275 and D22S1150 flanking the *CHEK2* gene used for haplotype analysis.

D22S1163		D22S689		D22S275		D22S1150	
Allele	Frequency	Allele	Frequency	Allele	Frequency	Allele	Frequency
141	0	202	0.067	158	0	213	0.019
143	0	206	0.019	160	0.028	215	0.057
145	0	210	0.134	162	0.163	217	0.038
147	0.201	214	0.201	164	0.144	219	0.192
149	0.269	218	0.211	166	0.201	221	0.038
151	0.230	222	0.048	168	0.259	223	0.451
153	0.115	226	0.019	170	0.096	225	0.028
155	0			172	0.009	227	0.019
157	0.105			174	0.076	229	0.038
159	0.076			176	0	231	0.028
161	0			178	0	233	0.057
163	0			180	0	235	0

Table S2. Haplotype analysis for microsatellite markers D22S1163, D22S689, D22S275 and D22S1150 in *CHEK2* c.499G>A carriers and non-carriers.

Sample ID	D22S1163	D22S689	D22S275	D22S1150
**F667**	143/159	202/210	168/168	223/231
**F1326**	143/149	210/210	168/168	223/223
**F2050**	143/151	210/218	168/168	215/223
**F2050a**	143/151	210/218	168/170	219/223
**F2276**	N.A.	N.A.	N.A.	N.A.
**F2469**	143/159	210/214	166/168	223/233
**F2469b**	143/143	206/210	168/168	223/231
**F2605**	143/151	210/218	162/168	223/229
**F2774**	143/147	210/214	164/168	223/235
**F3445**	143/153	210/214	168/168	223/233
**F3754**	N.A.	N.A.	N.A.	N.A.
**Common allele between carriers**	**143**	**210**	**168**	**223**
**Non-carriers**				
**F667a**	141/143	202/222	166/168	219/231
**F667b**	141/159	210/222	168/168	225/231
**F667c**	151/159	202/206	160/168	231/233
**F2469a**	157/159	214/222	162/166	215/233
**F2605a**	151/157	218/222	162/162	219/229
**F2605b**	151/159	202/218	162/166	219/229
**F2774a**	143/151	214/214	166/166	225/235
**F3445a**	149/153	214/218	166/166	219/233
**F3445b**	151/153	206/214	166/168	219/233

*Alleles segregating with the disease are highlighted in red.

Table S3. Haplotype analysis for microsatellite markers D22S1163, D22S689, D22S275 and D22S1150 in *CHEK2* c.549G>C carriers and non-carriers

Sample ID	D22S1163	D22S689	D22S275	D22S1150
**F459**	143/149	214/218	160/166	221/221
**F459a**	143/147	206/218	166/172	221/221
**F498**	143/159	214/218	166/170	213/221
**F498a**	143/149	214/218	166/170	219/221
**F1018**	143/149	210/218	164/166	221/233
**F1327**	143/143	218/222	166/166	215/221
**Common allele between carriers**	**143**	**218**	**166**	**221**
**Non-carriers**				
**459b**	149/159	210/218	160/162	223/223
**459c**	149/151	214/214	160/168	223/223
**498b**	151/157	214/214	170/170	213/219

*Alleles segregating with the disease are highlighted in red.

Table S4. Haplotype analysis for microsatellite markers D22S1163, D22S689, D22S275 and D22S1150 in *CHEK2* c.592+3A>T carriers and non-carriers

Sample ID	D22S1163	D22S689	D22S275	D22S1150
**F1313**	149/149	206/210	162/164	219/233
**F2482**	149/149	206/210	162/170	219/219
**F3247**	149/157	206/206	162/166	219/223
**F3710**	149/151	206/206	162/170	219/219
**F3710a**	149/151	206/206	162/162	219/219
**Common allele between carriers**	**149**	**206**	**162**	**219**
**Non-carriers**				
**2482a**	143/149	210/218	170/166	219/219
**2482b**	151/153	210/218	162/168	219/233
**2482c**	149/147	210/210	162/170	219/223

*Alleles segregating with the disease are highlighted in red.

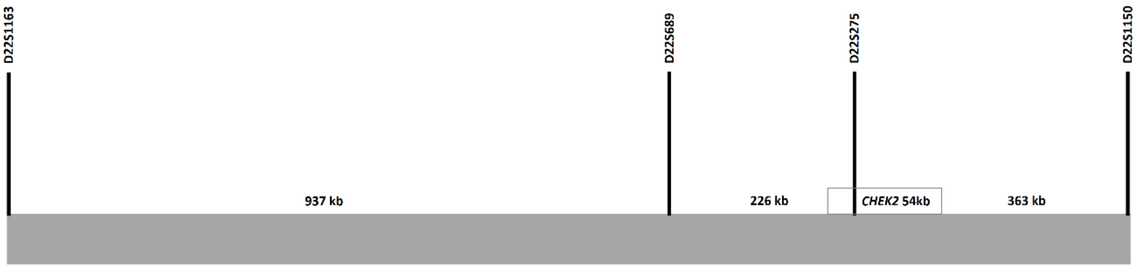

Figure S1. Physical map showing the microsatellite markers used for haplotype analysis relative to *CHEK2*, on chromosome 22.
